# East London Hospital for Children

**Published:** 1902-03-15

**Authors:** 


### EAST LONDON HOSPITAL FOR CHILDREN.

Owing to its out-of-the-way situation at Shadwell, amid miles of "mean streets," and also no doubt to the confidence felt by the subscribers in the excellence of the management, the East London Hospital for Children is not always able to secure the attendance of the legal minimum of 10 governors to form a quorum for its meetings, but on the occasion of the thirty-third annual court held on the 27th ultimo 13 were present at the opening, while two more arrived during the course of the proceedings.

### A Falling Revenue.

The ordinary receipts were £6,653, and the extraordinary receipts (legacies and festival dinner) £2,398. These show a decrease on the previous year's figures of £1,516, while the expenditure—£8,766 ordinary and £119 extraordinary—was £495 up, though the cost per occupied bed was £1 3s. 11¼d., as against £1 4s. in 1900. The report pointed out that the work, and consequently the expenditure of the hospital, was increasing yearly, and the board therefore noted with some apprehension that there was no equivalent increase of contributions, the annual subscriptions in particular having a tendency to fall off owing to new subscribers not being forthcoming to take the place of those removed by death during the year. Towards the latter end of 1901, the finances of the hospital were exceptionally straitened, but thanks to the prompt and generous response to the special appeal then issued the board were able to provide for all liabilities without resort to borrowing or other extreme measures. The annual festival dinner was held in April under the presidency of the Lord Mayor, and resulted in contributions amounting to £1,985. A concert in aid of the hospital realised £210, of which £106 was paid to the hospital, the balance being handed to the treasurer of the Ladies' Association.

### New Buildings and Extensions.

The scheme for increasing the accommodation of the hospital, details of which were set forth in the last annual and half-yearly reports, had occupied the close attention of the board, and a contract for £2,679 for the erection of the new mortuary and the laundry would shortly be signed. The cost of the equipment of the laundry with the addition of a steam disinfecting apparatus would be £985, making a total of £3,664 for carrying out this portion of the scheme. The urgent need of renewing these departments at once had been strongly impressed upon the board by the Council of the Prince of Wales's Hospital Fund, who, in 1900, made a special grant of £500 towards the cost of providing a new mortuary, and in December last awarded £250 towards the laundry, on the condition that the board put the work in hand in the present year. Including these grants, the sum at present available for building purposes was £1,622. The board urgently appealed for contributions to enable them to meet the balance of the cost of these works, and to provide the additional ward and domestic accommodation so badly needed. Mr. H. W. Trinder's resignation of the chairmanship of the Board of Management in November last was a matter of sincere regret. To the policy of Mr. Trinder must be attributed the increase of the invested property of the hospital, the income from which in 1892 was only £303, while in 1901 it amounted to £905.

### Seaside Branch.

The work done at the Princess Mary Convalescent Home at Bognor showed an increase, there having been 380 patients as compared with 316 in 1900. The daily average of occupied beds was 21·28 in 1901, as against 18·9 in the previous year. The average period of residence of the patients was 20·44 days, and the cost per week, per occupied bed, was 17s. 6d., being a reduction of 2d. per bed on the cost in 1900.

